# Overall Low Extended-Spectrum Cephalosporin Resistance but high Azithromycin Resistance in *Neisseria gonorrhoeae* in 24 European Countries, 2015

**DOI:** 10.1186/s12879-017-2707-z

**Published:** 2017-09-11

**Authors:** Michelle J. Cole, Gianfranco Spiteri, Susanne Jacobsson, Neil Woodford, Francesco Tripodo, Andrew J. Amato-Gauci, Magnus Unemo, Euro-GASP network, Alexander Indra, Virginie Maes, Tania Crucitti, Blaženka Hunjak, Tatjana Nemeth Blažić, Soteroulla Soteriou, Panayiota Maikanti-Charalambous, Despo Pieridou, Susan Cowan, Steen Hoffmann, Jevgenia Epstein, Jelena Viktorova, Ndeindo Ndeikoundam, Agathe Goubard, Peter Kohl, Susanne Buder, Viviane Bremer, Klaus Jansen, Eva Tzelepi, Vasileia Konte, Eszter Balla, Mária Dudás, Guðrún Sigmundsdóttir, Guðrún Svanborg Hauksdóttir, Derval Igoe, Brendan Crowley, Barbara Suligoi, Paola Stefanelli, Gatis Pakarna, Violeta Mavcutko, Christopher Barbara, Francesca Vella, Jackie Maistre Melillo, Alje Van Dam, Birgit Van Benthem, Ineke Linde, Hilde Kløvstad, Thea Bergheim, Slawomir Majewski, Beata Mlynarczyk-Bonikowska, Jacinta Azevedo, Maria José Borrego, Peter Pavlik, Peter Truska, Irena Klavs, Samo Jeverica, Julio Vazquez, Asunción Díaz, Inga Velicko, Magnus Unemo, Gwenda Hughes, Kate Templeton, Neil Irvine

**Affiliations:** 1 Antimicrobial Resistance and Healthcare Associated Infections (AMRHAI) Reference Unit, Public Health England, London, UK; 2 European Centre for Disease Prevention and Control, Stockholm, Sweden; 3 WHO Collaborating Centre for Gonorrhoea and other STIs, Örebro University, Örebro, Sweden

**Keywords:** Gonorrhoea, Treatment, Antimicrobial resistance, Ceftriaxone, Surveillance, European Gonococcal Antimicrobial Surveillance Programme (Euro-GASP), Europe, European Union (EU), European Economic Area (EEA)

## Abstract

**Background:**

Surveillance of *Neisseria gonorrhoeae* antimicrobial susceptibility in Europe is performed through the European Gonococcal Antimicrobial Surveillance Programme (Euro-GASP), which additionally provides data to inform the European gonorrhoea treatment guideline; currently recommending ceftriaxone 500 mg plus azithromycin 2 g as first-line therapy. We present antimicrobial susceptibility data from 24 European countries in 2015, linked to epidemiological data of patients, and compare the results to Euro-GASP data from previous years.

**Methods:**

Antimicrobial susceptibility testing by MIC gradient strips or agar dilution methodology was performed on 2134 *N. gonorrhoeae* isolates and interpreted using EUCAST breakpoints. Patient variables associated with resistance were established using logistic regression to estimate odds ratios (ORs).

**Results:**

In 2015, 1.7% of isolates were cefixime resistant compared to 2.0% in 2014. Ceftriaxone resistance was detected in only one (0.05%) isolate in 2015, compared with five (0.2%) in 2014. Azithromycin resistance was detected in 7.1% of isolates in 2015 (7.9% in 2014), and five (0.2%) isolates displayed high-level azithromycin resistance (MIC ≥ 256 mg/L) compared with one (0.05%) in 2014. Ciprofloxacin resistance remained high (49.4%, vs. 50.7% in 2014). Cefixime resistance significantly increased among heterosexual males (4.1% vs. 1.7% in 2014), which was mainly attributable to data from two countries with high cefixime resistance (~11%), however rates among men-who-have-sex-with-men (MSM) and females continued to decline to 0.5% and 1%, respectively. Azithromycin resistance in MSM and heterosexual males was higher (both 8.1%) than in females (4.9% vs. 2.2% in 2014). The association between azithromycin resistance and previous gonorrhoea infection, observed in 2014, continued in 2015 (OR 2.1, CI 1.2–3.5, *p* < 0.01).

**Conclusions:**

The 2015 Euro-GASP sentinel system revealed high, but stable azithromycin resistance and low overall resistance to ceftriaxone and cefixime. The low cephalosporin resistance may be attributable to the effectiveness of the currently recommended first-line dual antimicrobial therapy; however the high azithromycin resistance threatens the effectiveness of this therapeutic regimen. Whether the global use of azithromycin in mono- or dual antimicrobial therapy of gonorrhoea is contributing to the global increases in azithromycin resistance remains to be elucidated. The increasing cefixime resistance in heterosexual males also needs close monitoring.

## Background

Since the introduction of antimicrobial therapy of gonorrhoea, the rapid emergence and dissemination of antimicrobial resistance (AMR) in the causative agent, *Neisseria gonorrhoeae*, has been well-documented [1]. Due to the extraordinary ability of *N. gonorrhoeae* to rapidly and effectively develop AMR, combined multidisciplinary efforts are required to retain gonorrhoea as a treatable infection. These include: antimicrobial susceptibility surveillance of *N. gonorrhoeae*, including appropriate analysis of patient risk-group, the early identification of treatment failures, monitoring of antimicrobial usage, appropriate diagnostic testing strategies and evidence-based patient management [2, 3].

Surveillance of *N. gonorrhoeae* AMR in the European Union/European Economic Area (EU/EEA) is performed through the European Gonococcal Antimicrobial Surveillance Programme (Euro-GASP) and co-ordinated by the European Centre for Disease Prevention and Control (ECDC). Euro-GASP provides quality-assured antimicrobial susceptibility data, linked to patient clinical and epidemiological data. The programme aims to identify emergence of new AMR, monitor antimicrobial susceptibility and resistance over time in Europe, and to inform European [4] as well as national and other international gonorrhoea management guidelines. The European guidelines on the diagnosis and treatment of gonorrhoea currently recommend a single intramuscular dose of 500 mg of ceftriaxone plus a single oral dose of 2 g of azithromycin as empirical first-line dual antimicrobial therapy for all cases of urogenital and extra-genital gonorrhoea [4]. Euro-GASP documented a statistically significant increase in azithromycin resistance from 2013 (2.8%) to 2014 (7.9%) alongside a significant decrease in cefixime resistance from 4.7% to 2.0% [5]. Dual therapy with ceftriaxone and azithromycin has been recommended in Europe since 2012 as a strategy to delay the emergence and/or spread of ceftriaxone resistance [4]. However, the increasing azithromycin resistance documented in 2014 in Europe threatens this strategy, and in essence could leave ceftriaxone being used as monotherapy. Furthermore, the first failure (globally) to treat gonorrhoea with empirical dual antimicrobial therapy (250 mg ceftriaxone by single intramuscular dose plus 1 g of azithromycin by single oral dose) was recently reported in a male in the United Kingdom (UK) with pharyngeal gonorrhoea caused by a ceftriaxone- and azithromycin-resistant strain [6].

The present study describes the Euro-GASP antimicrobial susceptibility and resistance data from 24 European countries in 2015, linked to clinical and epidemiological data of the patients, and compares these results to Euro-GASP data from previous years, with particular focus on 2014.

## Methods

### European Gonococcal Antimicrobial Surveillance Programme (Euro-GASP)

*N. gonorrhoeae* isolates from 24 participating countries were included in the Euro-GASP in 2015 (Table 1). Isolates from consecutive patients were collected from September to November 2015 and antimicrobial susceptibility testing was performed using Etests (or other MIC gradient strips in some countries) or an agar dilution method (determination of minimum inhibitory concentration (MIC) or breakpoint technique) for ceftriaxone, cefixime, azithromycin, and ciprofloxacin as previously described [5]. Isolates from seven (29%) countries (Table 1) were tested centrally at Public Health England or Örebro University Hospital, Sweden. The remaining 17 (71%) countries followed a decentralised testing model, after fulfilling established quality criteria, where antimicrobial susceptibility testing was performed in their own laboratory (Table 1). All Euro-GASP laboratories participated in an annual external quality assessment (EQA) programme [7] to ensure comparability of antimicrobial susceptibility data. The antimicrobial susceptibility testing results were interpreted using EUCAST resistance breakpoints; cefixime/ceftriaxone MIC >0.12 mg/L, azithromycin MIC >0.5 mg/L, and ciprofloxacin MIC >0.06 mg/L [8]. The following clinical and/or epidemiological variables of the patients were collected and categorised: age (<25 years or ≥25 years), sexual orientation and gender (men who have sex with men (MSM), male heterosexuals and all women), previous gonorrhoea (yes or no), and concurrent chlamydial infection or no chlamydial infection.

**Table 1:** Resistance to cefixime, azithromycin and ciprofloxacin in *N. gonorrhoeae* isolates from 24 EU/EEA countries, 2015

Country	No. of isolates tested	Resistance						Method of testing
		Cefixime		Azithromycin		Ciprofloxacin		
		No.	%	No.	%	No.	%	
Austria	61	0	0.0%	2	3.3%	40	65.6%	Decentralised – Etest
Belgium	99	11	11.1%	3	3.0%	49	49.5%	Decentralised – AD
Croatia	8	0	0.0%	0	0.0%	3	37.5%	Centralised – Etest
Cyprus	3	0	0.0%	0	0.0%	2	66.7%	Decentralised – Etest
Denmark	110	0	0.0%	3	2.7%	34	30.9%	Decentralised – Etest
Estonia	18	0	0.0%	0	0.0%	5	27.8%	Centralised – Etest
France	105	0	0.0%	6	5.7%	44	41.9%	Decentralised – Etest
Germany	109	2	1.8%	2	1.8%	67	61.5%	Centralised – BKP/Etest
Greece^a^	100	11	11.0%	22	22.0%	77	77.0%	Decentralised – Etest
Hungary	64	1	1.6%	3	4.7%	34	53.1%	Centralised - BKP/Etest
Iceland	14	0	0.0%	0	0.0%	4	28.6%	Decentralised – Etest
Ireland	110	1	0.9%	20	18.2%	50	45.5%	Decentralised – Etest
Italy	100	0	0.0%	2	2.0%	71	71.0%	Decentralised – Etest
Latvia	9	0	0.0%	0	0.0%	1	11.1%	Centralised – Etest
Malta	29	0	0.0%	4	13.8%	19	65.5%	Decentralised – Etest
Netherlands	200	0	0.0%	8	4.0%	74	37.0%	Decentralised – Etest
Norway	110	1	0.9%	4	3.6%	64	58.7%^b^	Decentralised – AD
Poland	56	0	0.0%	3	5.4%	32	57.1%	Centralised – Etest
Portugal	110	0	0.0%	19	17.3%	41	37.3%	Decentralised – Etest
Slovakia	104	4	3.8%	2	1.9%	56	53.8%	Centralised – Etest
Slovenia	109	0	0.0%	0	0.0%	38	34.9%	Decentralised – Etest
Spain	167	4	2.4%	5	3.0%	109	65.3%	Decentralised – Etest
Sweden	100	0	0.0%	14	14.0%	45	45.0%	Decentralised – Etest
UK	239	1	0.4%	30	12.6%	95	39.7%	Decentralised – AD/Etest
Total:								
Cefixime	2132	36	1.7%					
Ciprofloxacin	2133					1054	49.4%	
Azithromycin	2134			152	7.1%			
95% CI			1.2–2.3		6.1–8.3		47.3–51.5	

*EU/EEA* European Union/European Economic Area, *No.* Number, *Etest* minimum inhibitory concentration (MIC) gradient strips to determine the MIC of an antimicrobial (mostly Etests, but also some other MIC gradient strips were used in some countries), *AD* agar dilution method to determine the MIC of an antimicrobial, *BKP* Breakpoint agar dilution method, *CI* confidence interval of the mean %

^a^Only one (0.05%) ceftriaxone resistant isolate was identified in Euro-GASP in 2015 (in Greece; MIC = 0.25 mg/L)

^b^Calculated from 109 isolates with ciprofloxacin results

### Statistical analysis

Statistical analysis was performed in STATA v13.1 (StataCorp LP, TX, USA) and included the Z-test to establish significance of changes in the proportion of isolates with AMR between 2014 and 2015. Patient variables associated with AMR were established using univariate and multivariable logistic regression analyses and associations were expressed as odds ratios (OR) with 95% confidence intervals (CI). A Pearson χ^2^-test was used to test whether these odds ratios were significantly different from one. For small cell numbers, Fisher’s exact test was used. A *P*-value of <0.05 was considered to indicate significance for all tests.

## Results

A total of 2134 *N. gonorrhoeae* isolates were examined in 2015. Most (81.8%) isolates were collected from male patients. The age of the patients ranged from less than one year to 79 years, with a median age of 29 years. Overall, 29.5% of patients were under 25 years of age and males (median age 30 years) were significantly older than women (median age 24.5 years) (*p* < 0.001). The anatomical site of specimen collection was mainly urogenital (72.9%), followed by rectal (13.5%) and pharyngeal (8.7%). Among cases with information on previous diagnosis of gonorrhoea (42.0%) and concurrent STI (37.8%), 17.5% had previously been diagnosed with gonococcal infection and 19.0% had a concurrent *Chlamydia trachomatis* infection. Among cases with known sexual orientation and gender (68.5%), 55% were heterosexual men (29%) or women (26%), and 45% were MSM.

**Fig. 1:**
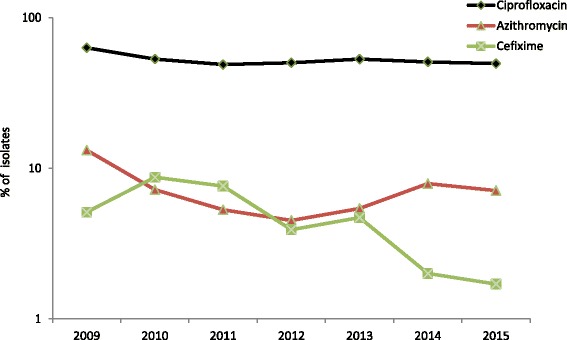
Trends in cefixime, azithromycin, and ciprofloxacin gonococcal resistance in the EU/EEA, 2009*–*2015. Note: logarithmic scale on y-axis. Number of ceftriaxone resistant isolates (MIC > 0.125 mg/L); 2009 and 2010 (*n* = 0), 2011 (*n* = 10), 2012 (*n* = 3), 2013 (*n* = 7), 2014 (*n* = 5), and 2015 (*n* = 1)

**Fig. 2:**
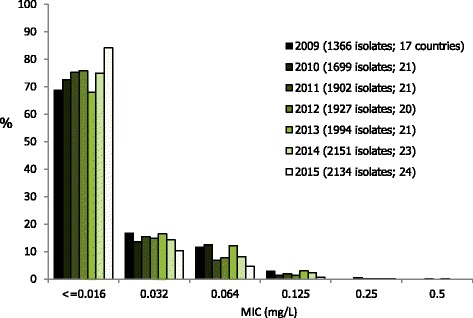
Ceftriaxone MIC distribution for *N. gonorrhoeae* isolates in the EU/EEA, 2009–2015

The antimicrobial susceptibility testing results are summarised in Table 1. Cefixime resistance was detected in 1.7% (36 out of 2132) of isolates (Table 1, Fig. 1) representing a stable overall resistance level compared with 2014 (2.0%, 42/2101) (*p* = 0.45). Cefixime resistance was detected in nine (37.5%) countries (10 (43%) countries in 2014 and 13 (62%) in 2013), and in these countries the cefixime resistance levels ranged from 0.4% in the UK to ≥11.0% in Belgium and Greece (Table 1). There were seven isolates (0.3%; from Greece (*n* = 5), Slovakia (*n* = 1) and Spain (*n* = 1)) with cefixime MICs of 0.5 mg/L compared with three (0.1%) isolates in 2014; the proportion of highly susceptible isolates (cefixime MIC ≤0.016 mg/L) continued to increase (from 61% in 2013 to 71% in 2014 and 75% in 2015). Only one (0.05%) isolate displayed ceftriaxone resistance compared with five (0.2%) isolates in 2014 and seven (0.4%) in 2013. This single ceftriaxone-resistant isolate (MIC = 0.25 mg/L) in 2015 was from Greece and additionally had intermediate susceptibility to azithromycin (MIC = 0.5 mg/L). There were an additional 16 (0.7%) isolates with ceftriaxone MICs of 0.125 mg/L (i.e. on the breakpoint for resistance) and nine (0.4%) of these isolates were also resistant to azithromycin. The MIC distribution for ceftriaxone in 2015, compared with 2009–2014, showed a higher proportion of more susceptible gonococcal isolates (MIC ≤ 0.016 mg/L) and a decreased proportion of isolates with higher MICs (0.032 mg/L to 0.125 mg/L) (Fig. 2).

**Fig. 3:**
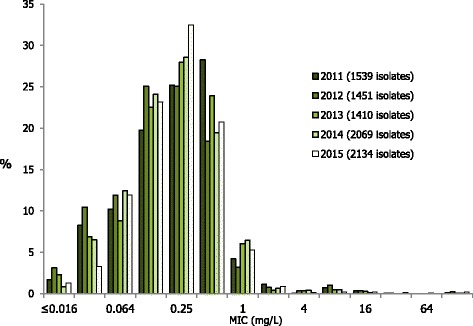
Azithromycin MIC distribution for *N. gonorrhoeae* isolates in the EU/EEA, 2011–2015

The overall resistance to azithromycin was 7.1% (152/2134 isolates), which represented the first break from the increasing trend in azithromycin resistance that has been observed in Euro-GASP data since 2012, although the decrease when compared with 2014 (7.9%) was not significant (*p* = 0.35). Azithromycin resistance ranged from 0% (Croatia, Cyprus, Estonia, Iceland, Latvia and Slovenia; of which all except Slovenia examined ≤18 isolates) to 22% in Greece (Table 1). Five (0.2%) isolates displayed high-level resistance to azithromycin (HLAziR (MIC ≥ 256 mg/L); Ireland (*n* = 3), Norway (*n* = 1) and UK (*n* = 1)) compared with one (0.05%) in 2014. The MIC distribution for azithromycin in 2015, compared with 2011 (when MIC determination was introduced) to 2014, showed an increasing proportion of resistant isolates (MICs >0.5 mg/L). However, most (87%) of the resistant isolates in 2015 had a low-level resistance (MICs ≤2 mg/L), which was similar to previous years (Fig. 3). Five isolates (Greece (*n* = 2), Hungary (*n* = 1), Slovakia (*n* = 1) and UK (*n* = 1)) were resistant to both azithromycin and cefixime. Ciprofloxacin resistance was detected in 49.4% (1054/2133) of isolates in 2015, which was similar to resistance levels observed in 2014 (50.7%) and 2013 (53%) (Fig. 1).

In 2015, cefixime resistance significantly increased among heterosexual males (4.1% vs. 1.7% in 2014, *p* = 0.045) whereas it decreased among females (1%; 2.5% in 2014, *p* = 0.16, Fisher’s Exact test) and MSM (0.5%; 1.2% in 2014, *p* = 0.21, Fisher’s Exact test). Cefixime resistance was significantly associated (*p* < 0.01) with heterosexual males in 2015 compared with females (OR = 0.3, CI = 0.08–0.8) and MSM (OR = 0.1, CI = 0.03–0.4), whereas no patient characteristics were significantly associated with cefixime resistance in 2014. Higher levels of azithromycin resistance were also detected in 2015 in male heterosexuals (8.1%; 8.9% in 2014) and MSM (8.1%; 9.9% in 2014) compared to in females (4.9%; 2.2% in 2014), but this difference was not significant (male heterosexuals *p* = 0.07 and MSM *p* = 0.06). The association between azithromycin resistance and previous gonorrhoea infection first observed in 2014 continued in 2015 (OR 2.1, CI 1.2–3.5, *p* < 0.01). The only patient association with ciprofloxacin resistance in 2015 was being a heterosexual male compared with MSM (OR 1.9, CI 1.5–2.4, *p* < 0.01), in contrast to 2014 when higher age (≥25 years) and the absence of a concurrent chlamydial infection were also associated with ciprofloxacin resistance.

## Discussion

The 2015 Euro-GASP surveillance data, examining AMR gonococci in 24 (77%) EU/EEA countries, showed that the growing increase in azithromycin resistance documented since 2012 appears to have stalled, and the resistance levels to ceftriaxone and cefixime remain stable and low. This low level of resistance to third-generation extended-spectrum cephalosporins appears to reflect the situation documented from well-established national surveillance programmes in many geographic settings and is likely a consequence of the effectiveness of the current first-line dual antimicrobial therapy in combination with appropriate diagnostics and patient management. For example, in the USA resistance to cefixime (MIC >0.125 mg/L) and decreased susceptibility to ceftriaxone (MIC ≥0.125 mg/L, as described by the Centers for Disease Control and Prevention (CDC), Atlanta, USA) was documented in 0.8% and 0.1% of isolates, respectively, in 2014 [9]. Low levels of resistance to cefixime and decreased susceptibility to ceftriaxone (1.1% and 2.7%, respectively) was also documented in Canada in 2014 [10] using the identical breakpoints as CDC [9], and no ceftriaxone resistance (MIC >0.12 mg/L) was reported from Australia in 2014 [11]. No ceftriaxone resistance (MIC >0.12 mg/L) was recently documented in Fukuoka, Japan [12], although the high, but decreasing, proportion of cefixime resistance (26% in 2013) is still of concern. Unfortunately, a higher level of ceftriaxone resistance was documented in China; 4.4% in 2012 to 2013 [13].

Despite positive results for the extended-spectrum cephalosporins, the high rates of azithromycin resistance documented by Euro-GASP threaten the effectiveness of the recommended dual antimicrobial therapy, and increasing azithromycin resistance is also reported globally. An analysis of azithromycin susceptibility in *N. gonorrhoeae* from 2005 to 2013 in the USA [14] revealed no temporal trend in azithromycin reduced susceptibility/resistance (MIC > 2 mg/L), which ranged from 0.3% to 0.6% during the examined years. This suggested that there was no impact on the level of azithromycin resistance by the use of dual antimicrobial therapy in the USA (initiated in 2010) and prompting the authors to state “*it is possible that we overestimated the capacity of N. gonorrhoeae to acquire azithromycin resistance*”; a reasonable statement especially as azithromycin is the most commonly prescribed antimicrobial agent in the USA [15]. However, the 2014 data for the USA [9] revealed a substantial increase in azithromycin resistance to 2.5%, which is the highest level since 1992 when testing for azithromycin resistance started. The authors also noted that azithromycin resistance was most prevalent in the mid-west of the USA, suggesting ‘home-grown’ resistance within the USA, as opposed to the traditional importation of resistant gonococcal strains in the western part of the USA from South East Asia and subsequent clonal national spread as seen with ciprofloxacin resistance and penicillinase-producing *N. gonorrhoeae* (PPNG) [9]. Again, a similar picture was observed in Canada during the same time period; an increase in azithromycin resistance from 0.4% in 2011 to 3.3% in 2014 [10]. In Fukuoka, Japan, azithromycin resistance (MIC >0.5 mg/L) increased from 1.8% in 2010 to 22.6% in 2013 [12], which in part was attributed to the use of 2 g of azithromycin (extended-release formulation) as monotherapy for gonorrhoea. The level of azithromycin resistance has also been increasing in Australia; from 1.1%–1.3% in 2011–2012 to 2.4% in 2014 [11]. National studies in European countries have also observed increases in azithromycin resistance, e.g. from 1% in 2014 to 9.8% in the UK in 2015, although this increase was partly due to a change in the agar medium used for antimicrobial susceptibility testing [16]. The five HLAziR isolates documented in Euro-GASP in 2015 represent the highest number since the beginning of the Euro-GASP surveillance and reports of outbreaks or sporadic detection of HLAziR globally [10, 11, 16–20] are of obvious concern. The mechanisms of the azithromycin resistance in the 2015 Euro-GASP isolates have not been investigated. However, as in previous studies the high-level azithromycin resistant isolates likely contain an A2059G (*Escherichia coli* numbering) mutation in three or four of the 23S rRNA gene alleles, whereas the isolates with lower level of azithromycin resistance comprise the C2611T mutation in 23S rRNA and/or mutations in the promoter or coding sequence of mtrR [1, 20].

A recent study from Guangzhou, China reported that 32.5% of isolates with azithromycin resistance (MIC ≥1.0 mg/L) also had decreased susceptibility to ceftriaxone (MIC ≥0.125 mg/L) [21]. In addition, reports from Ontario, Canada [22] and Hawaii [19] have described clonal spread of isolates with both azithromycin resistance and reduced susceptibility to cephalosporin. Clonal spread of isolates with azithromycin and ceftriaxone resistance has been previously documented in some *N. gonorrhoeae* multi-antigen sequence typing (NG-MAST) ST1407 isolates [19, 23, 24], and spread of these types of clones is of most concern to the global gonococcal surveillance community and healthcare clinicians.

Whether the global use of azithromycin in mono- or dual antimicrobial therapy for gonorrhoea is contributing to the increasing azithromycin resistance is difficult to establish in the absence of data comparing the impact of the different regimens on the susceptibility profile of the circulating gonococcal population. The widespread use of ceftriaxone in combination with azithromycin for empirical first-line treatment of all cases of uncomplicated gonorrhoea, as currently recommended in the European gonorrhoea management guideline [4] and similar therapeutic regimens [25, 26], has likely maintained gonorrhoea a treatable infection for the present. Nevertheless, it remains to be seen if the combination with azithromycin or the increased dosage of ceftriaxone that accompanied the dual antimicrobial therapy implementation in many regimens, has contributed most to the currently low level of resistance to extended-spectrum cephalosporins. Even though ceftriaxone and cefixime have been shown to have comparable efficacies for fully susceptible gonococcal isolates [27–29] and free-drug concentration time periods that exceed MIC (*f*T > MIC) in most gonococcal strains [30], cefixime may be more prone to promote resistance development than ceftriaxone. This is supported by the globally documented decreasing cefixime resistance which followed the removal of cefixime from recommended first-line empirical therapy, and the higher number of treatment failures with cefixime versus ceftriaxone [1].

The increasing cefixime resistance observed in heterosexual males (4.1%) was mainly a result of overall high resistance rates in the isolates from two countries; Belgium (11.1%, all isolates MIC = 0.25 mg/L) and Greece (11%; 6 isolates MIC = 0.25 mg/L and 5 isolates MIC = 0.5 mg/L). Greece did not submit any isolates from women and therefore any high cefixime resistance among women from Greece would not have contributed to the overall European rate. However the lower proportion of isolates from women (18.2%) compared with men (81.8%) may contribute overall to an underestimation of resistance in women and in general in heterosexual networks, as is the case in all GASPs, particularly in the USA where isolates from females are not included [14]. The Euro-GASP collaborators in Greece and Belgium have stated that cefixime, which could more effectively select for resistance, is not frequently used as first-line treatment for gonorrhoea in their countries. Furthermore, the overall level of cefixime resistance in MSM was low (0.5%), which might be because cefixime resistance was previously frequent in this group and, consequently, dual antimicrobial therapy (ceftriaxone plus azithromycin) or at a minimum ceftriaxone has been used to treat gonorrhoea more frequently in this group. Unfortunately, data on prescribed treatment in Euro-GASP are not available pre-2013, and the level of reporting for this variable was low in 2014 (18.6%) and 2015 (36.5%) [5].

The frequent use of azithromycin for empirical treatment of non-gonococcal urethritis may be driving the higher azithromycin resistance in men. However, the use of azithromycin to treat *C. trachomatis* and *Mycoplasma genitalium* infections in both genders should also be considered. It should be noted that the two countries with the highest azithromycin resistance, Greece (22%) and Ireland (18%), both submitted isolates predominantly from men and that MICs for the majority of azithromycin-resistant isolates were just above the resistance breakpoint. Results of azithromycin susceptibility tests vary with changes in agar medium composition, pH and incubational parameters such as CO_2_ levels [31] so even though MICs in the laboratories are comparable, slight technical differences may increase or decrease MICs by one or more two-fold dilution steps and affect the clinical interpretation.

Improving the representativeness of Euro-GASP by, for example, including more isolates from females, increasing its geographic representativeness and increasing the completeness of reporting of patient variables are part of the ongoing Euro-GASP work programme in order to reduce country biases as far as possible. However, due to the heterogeneity of healthcare systems across Europe, a ‘one-size-fits-all approach’ may never be possible and differences in isolate collection, selection and geographical representativeness may be an inherent limitation of any large multi-country sentinel surveillance programme such as Euro-GASP.

## Conclusions

Even though ceftriaxone resistance is still low and the MIC distribution is currently showing little signs of concern, the 2015 azithromycin data emphasize the need to continue expanding and improving Euro-GASP and other GASPs as emphasised in the European and WHO action plans to detect and prevent the emergence and spread of AMR in *N. gonorrhoeae* [2, 3]. The increasing global resistance to azithromycin is of major concern and threatens the future effectiveness of the recommended dual antimicrobial therapies introduced in many well-resourced settings [4, 25, 26, 32–34]. We should be particularly alert to the spread of ceftriaxone and azithromycin co-resistance and continue to monitor closely for treatment failures, such as the recently reported first treatment failure globally to the recommended dual antimicrobial therapy regimen [6].

## Abbreviations

AD: Agar dilution method
AMR: Antimicrobial resistance
BKP: Breakpoint agar dilution method
CDC: Centers for Disease Control and Prevention
CI: Confidence intervals
ECDC: European Centre for Disease Prevention and Control
EEA: European Economic Area
EU: European Union
Euro-GASP: European Gonococcal Antimicrobial Surveillance Programme
*f*T: Free-drug concentration time
HLAziR: High-level resistance to azithromycin
MIC: Minimum inhibitory concentration
NG-MAST: *Neisseria gonorrhoeae* multi-antigen sequence typing
No.: Number
OR: Odds ratio
PPNG: Penicillinase-producing *Neisseria gonorrhoeae*
UK: United Kingdom
